# Systematic Review and Meta-Analysis on Incidence of Altered Sensation of Mandibular Implant Surgery

**DOI:** 10.1371/journal.pone.0154082

**Published:** 2016-04-21

**Authors:** Chia-Shu Lin, Shih-Yun Wu, Hsin-Yi Huang, Yu-Lin Lai

**Affiliations:** 1 Department of Dentistry, School of Dentistry, National Yang-Ming University, Taipei, Taiwan; 2 Division of Family Dentistry, Department of Stomatology, Taipei Veterans General Hospital, Taipei, Taiwan; 3 Biostatistics Task Force, Taipei Veterans General Hospital, Taipei, Taiwan; 4 Division of Endodontics and Periodontology, Department of Stomatology, Taipei Veterans General Hospital, Taipei, Taiwan; Georgia Regents University, College of Dental Medicine, UNITED STATES

## Abstract

Altered sensation (including paresthesia, dysesthesia and hypoesthesia) after mandibular implant surgery may indicate transient or permanent injury of the inferior alveolar nerve and the mental branch, and considerably lower patients’ satisfaction about the therapy. Previous studies have shown a great degree of variability on the incidence of altered sensation. We here reported the incidence of altered sensation after mandibular implant surgery based on a meta-analysis of 26 articles published between 1990.1.1 and 2016.1.1. Study quality and risk of bias was assessed and the studies with a lower score were excluded in the meta-analysis. Data synthesis was performed using the logistic-normal random-effect model. The meta-analyses revealed that the short-term (10 days after implant placement) and long-term (1 year after implant placement) incidence was 13% (95% CI, 6%-25%) and 3% (95% CI, 1%-7%), respectively. (2) For the patients who initially reported altered sensation, 80% (95% CI, 52%-94%) of them would return to normal sensation within 6 months after surgery, and 91% (95% CI, 78%-96%) of them would return to normal sensation one year after surgery. We concluded that dentist-patient communication about the risk of altered sensation is critical to treatment planning, since the short-term incidence of altered sensation is substantial (13%). When a patient reports altered sensation, regular assessment for 6 months would help tracing the changes of symptoms. In terms of long-term follow-up (1 year after surgery), the incidence is much lower (3%) and most patients (91%) would return to normal sensation.

## Introduction

One of the most disturbing complications associated with implant surgery is post-operative altered sensation, including paresthesia, dysesthesia and hypoesthesia [1,2]. For mandibular implant surgery, altered sensation may be associated with an injury of the inferior alveolar nerve (IAN) and the mental branch. Persistent altered sensation may indicate neurosensory dysfunction or IAN neuropathy [3,4]. Even transient altered sensation, such as numbness or tingling, may considerably lower patients’ satisfaction about implant surgery and, occasionally, result in medico-legal problems [2,5]. Therefore, the occurrence of post-operative altered sensation, such as paresthesia, has been widely considered as one of the success criteria of implant surgery [6,7], and recently, the diagnosis and prevention of altered sensation have been extensively discussed in the literature [1,8,9]. The occurrence of altered sensation has been investigated in previous reviews, as one of the general complications of implant surgery [10–12]. However, an estimation of the incidence of altered sensation after mandibular implant surgery, based on systematic review and meta-analysis, has not been reported. Based on clinical literature, the incidence of altered sensation varied considerably across studies, from 0.13% [13] to 37% [14]. Therefore, researchers can only estimate the incidence with a great degree of variability, such as 0–15% [8] or 0–40% [15]. In addition, the recovery rate of altered sensation, i.e. the proportion of the patients with altered sensation returning to normal sensation, has still remained unclear. More precise estimates of the incidence and the recovery rate of altered sensation, based on meta-analysis, would contribute to the risk assessment of surgery and patient-dentist communication, during treatment planning. Finally, elderly patients usually have a severely atrophic ridge, with a short distance between the ridge and the IAN [16,17]. The influences of patients’ age and the degree of ridge atrophy on the incidence of altered sensation have not been fully assessed.

The current systematic review and meta-analysis aimed to estimate the incidence of altered sensation after mandibular implant surgery. Specifically, we focused on the following aims:

1. First, we estimated the short-term (assessed postoperatively or within 10 days after implant placement) and long-term (assessed 1 year after implant placement) incidence of altered sensation after mandibular implant surgery.
2. Secondly, we estimated the recovery rate of initial altered sensation, i.e., the proportion of the patients with altered sensation returning to normal sensation.

## Materials and Methods

The review and meta-analysis were performed according to the Preferred Reporting Items for Systematic Reviews and Meta-Analyses for Protocols (PRISMA-P, see S1 Table for the checklist) [18]. The review protocol has been registered based on PROSPERO (http://www.crd.york.ac.uk/PROSPERO) (CRD42015016250) and the review was performed accordingly.

### Eligibility Criteria

We selected the original research articles based on the following criteria:

- Participants: The participants included in the review were the adults who were eligible for implant surgery, according to the criteria established in the individual studies. All participants received mandibular implant surgery.
- Outcomes: The individual studies should report the number of patients who showed altered sensation. Our primary outcome was the incidence of altered sensation, defined as the proportion (i.e., the event rate) of the number of patients who showed altered sensation to the number of total patients being assessed after the surgery. ‘Altered sensation’ was identified if one of the following conditions was reported: dysesthesia, paresthesia (including changes in sensation, such numbness or tingling), hypoesthesia, anesthesia, or sensory changes as assessed by sensory testing [9]. The outcomes were further categorized according to the time point of clinical assessment. We considered the altered sensation recorded postoperatively or within 10 days after implant placement to be *short-term*, and the altered sensation recorded 1 year after implant placement to be *long-term*. Additionally, we considered the altered sensation recorded 3–6 months after implant placement to be *intermediate-term*, based on the model of nerve injury [19]. The articles that reported altered sensation during the period other than the previous time points would still be included. The model predicts that spontaneous healing would occur within 4 months, if nerve sheet is not damaged by trauma (i.e., neuropraxia and axonotmesis) [19,20].

### Search Strategy

We performed a computerized search of original research articles on the electronic database MEDLINE and The Cochrane Library, with the keywords related to implant surgery and altered sensation. It should be noted that, because the term ‘altered sensation’ may refer to different experiences in studies, we included a variety of keywords related to altered sensation in our search, including allodynia, hyperalgesia, dysesthesia, paresthesia, sensory disturbance, numbness etc. (see Table 1 for the complete search strategy). Additionally, a manual search was performed by screening the reference list from the previous reviews on implant-related altered sensation [12,21,22]. The findings from the computerized and the manual search were pooled for subsequent selection. Search was limited to the journal articles published in English, during the period from 1990 Jan.1 to 2016 Jan. 1. Case reports were excluded.

**Table 1:** Search Strategy.

(A) Computerized search on PubMed/MEDLINE		
Search	Query	Items found
#1	Search (dental OR oral OR "inferior alveolar nerve") AND (implant OR implants)	42409
#2	Search ("altered sensation" OR allodynia OR hyperalgesia OR dysaesthesia OR paresthesia OR (sensory disturbance*) OR numbness OR (neurosensory disturbance*) OR "psychological impact" OR "nerve injury" OR neuropathic)	63147
#3	Search (#1 AND #2)	314
#4	Search (#3 NOT "case reports"[ptyp])	266
#5	Search (#3 NOT "case reports"[ptyp]) Filters: Publication date from 1990/01/01 to 2016/01/01; Humans; English	182
(B) Computerized search on Cochrane Library		
	Publication Year from 1990 to 2016, in Trials, Methods Studies and Technology Assessments	
Search	Query	Items found
#1	Search (dental OR oral OR "inferior alveolar nerve") AND (implant OR implants)	2730
#2	Search ("altered sensation" OR allodynia OR hyperalgesia OR dysesthesia OR paresthesia OR "sensory disturbance" OR numbness OR "neurosensory disturbance" OR "psychological impact" OR "nerve injury")	4716
#3	Search (#1 AND #2)	19
#4	Publication year from 1990/01/01; English	17

### Study Selection

**Fig 1:**
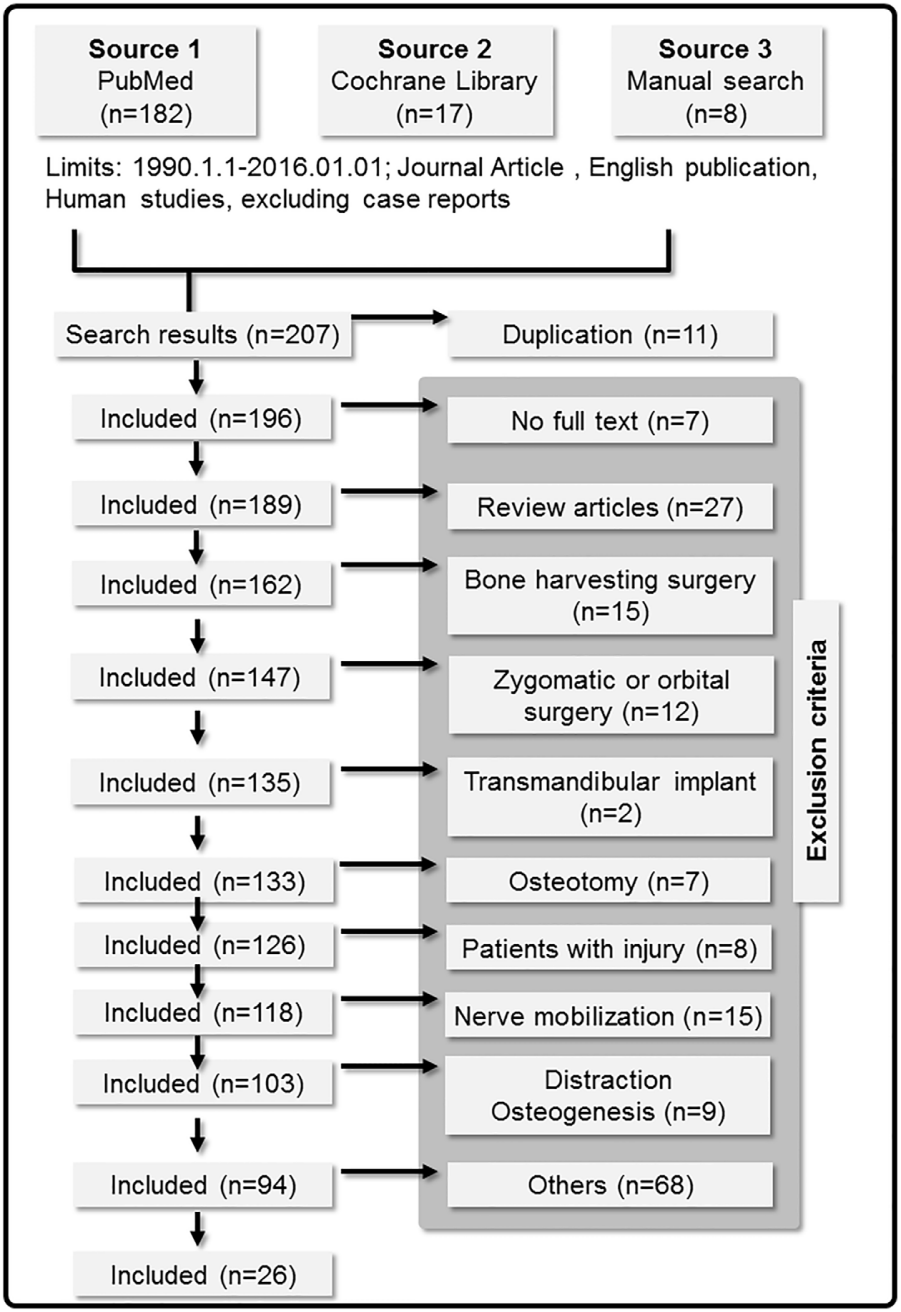
Flow diagram describing the process of review and study selection.

We manually screened the initially found articles by excluding those who focused on the following topics: transmandibular implant surgery, zygomatic or orbital implant surgery, nerve transposition, nerve lateralization, distraction osteogenesis, osteotomy for orthodontic purposes, or the bone-harvesting surgery without implant placement. The articles that no full text can be retrieved, according to PubMed, were also excluded (Fig 1). Review articles were excluded. Two authors (CSL and SYW) independently conducted literature search and the assessment of eligibility and study selection, primarily according to the title and the abstract of the selected articles. Decisions were made by a consensus between the authors. The selected articles were sorted using Endnote (Thomson Reuters, New York, NY, USA).

### Data Collection and Extraction of Data Items

Printed or electronic full text of all the included studies was retrieved. The following data items were manually extracted:

(A) Demographic and clinical characteristics (5 items, see Table 2), including (1) the design of the study (e.g., a randomized control trial of a retrospective/prospective cohort study), (2) the number of patients (total and by each sex), (3) the mean or the range of patients’ age, (4) patients’ ridge condition, focusing on alveolar bone height, the ridge-to-IAN canal distance (RCD), the Cawood’s class of mandible atrophy [23], and the Lekholm and Zarb’s class of jaw bone quality [24], (5) the surgical methods for ridge augmentation.

**Table 2:** Demographic and Clinical Characteristics of the Included Studies.

ID	Source	Design	#Patient (Total/ Female/Male)^1^	Age	Ridge condition^2^	Augmentation procedure
1	Boven 2014[25]	RS	40/33/7	61	Height 8.9±2.2 Atrophy VI	Illiac crest onlay bone graft
2	Kütük 2013[54]	RS	55/NR	NR	RCD ≥5	NR
3	Geckiki 2011[55]	RS	23/16/7	63	NR	NR
4	Bormann 2010[33]	NR	13/8/5	56	RCD >4	Inter-positional autograft
5a	Felice 2009a[36]^3^	RCT	15/11/4	56	RCD 5–7	Bio-Oss
5b	Felice 2009a[36]^3^	RCT			RCD 5–7	NR
6a	Felice 2009b[35]	RCT	30/15/15	55	RCD 7–8	Bio-Oss
6b	Felice 2009b[35]	RCT	30/23/7	56	RCD 7–8	NR
7	Burnstein 2008[37]	NR	20/NR	NR	RCD <10	NR
8	Vazquez 2008[13]	PS	1527/890/637	53	NR	NR
9	Abarca 2006[52]	NR	65(58)/30/35	58	NR	NR
10	van der Meij 2005[38]	RS	17/13/4	56	Height 8.5 Atrophy VI	Illiac crest onlay bone graft
11	Visser 2005[56]	RS	60/39/21	54.9	Height 12–18 Atrophy V-VI Quality 3/2.7^5^	NR
12	Frei 2004[57]	PS	50/30/20	54	Height 13.9±2.7	NR
13	Tortamano- Neto 2004[58]	PS	10(6)/5/5	28–61	Sufficient bone volume	NR
14	Walton 2000[59]	PS	75/47/28	63	NR	NR
15	Bartling 1999[41]	PS	94/43/51	NR	NR	NR
16	Allen 1997[40]	RS	60/ NR	NR	NR	NR
17	Friberg 1997[60]	PS	103/54/49	59	NR	NR
18	Wismeijer 1997[28]^4^	PS	110(105)/34/76	51.5	NR	NR
19	Batenburg 1994[61]	RS	57/40/17	58	Atrophy V-VI	NR
20	Ellies 1993[21]	RS	112(87)/58/29	58	NR	NR
21	Ellies 1992[14]	RS	212/155/57	57	NR	NR
22	Johns 1992[26]	PS	133/79/54	57	Quality 1–3^6^	NR
23	Åstrand 1991[62]	NR	23/15/8	57.7	NR	NR
24	Kiyak 1990[53]	PS	39(27)/31/8	57.5	NR	NR
25	van Steenberghe 1990[27]	PS	159/92/67	41–60	Quality 3–4^7^	NR
26	Zarb 1990[63]	NR	46/36/10	NR	NR	NR

NR: not reported; PS, prospective cohort study; RCD: ridge-to-canal distance; RCT, randomized control trial; RS, retrospective cohort study.

^1^The number in the brackets denotes the number of responders.

^2^Degree of bone atrophy was assessed by Cawood's classification; degree of bone quality was assessed by Lekholm and Zarb's classification; RDC and height is measured in mm.

^3^The study adopted a within-subject split-mouth design.

^4^Mean age was calculated based on Table 1 of the study.

^5^Bone quality was assessed respectively for two study groups.

^6^Most implants were placed with bone quality between 1 and 3, based on Table 5 of the study.

^7^The bone quality of 57% of the mandibles was of grade 3 or 4.

(B) Methodological characteristics (4 items, see Table 3), including (1) the length and diameter of implant (2) pre-operative imaging examination, focusing on the use of panoramic radiograph or tomograph/computed tomography, (3) the methods for assessing altered sensation, and (4) the specific experience of altered sensation (e.g., paresthesia or hypoesthesia) being assessed.

**Table 3:** Methodological characteristics of the included studies.

ID	Implant (Length/ Diameter) *	Preoperative imaging examination	Assessment of altered sensation	Experience of altered sensation
1	>10/4.1	NR	light touch test, pin-prick test	sensory change
2	NR	Pano	mechanoceptive test	pain (tingling / throbbing)
3	NR	Pano	NR	pain, paresthesia
4	6-12/3.3–4.5	Pano, DVT	NR	hypoesthesia
5a	10-13/4	CT	NR	paresthesia
5b	5/6	CT	NR	paresthesia
6a	>10/NR	CT	NR	paresthesia
6b	7/NR	CT	NR	paresthesia
7	>5/NR	NR	NR	NR
8	6-12/NR	Pano	2-point discrimination, pin-prick test	hypoesthesia, anesthesia, paresthesia
9	NR	NR	questionnaire, 2-point discrimination, light touch test, thermal test	neurosensory disturbance
10	13-15/3.8–4.5	Pano	NR	neurosensory disturbance
11	11-15/NR	NR	NR	NR
12	8-12/4.1–4.8	Pano, spiral tomograh	NR	paresthesia
13	10/4.1	NR	NR	pain, paresthesia
14	NR	Pano	questionnaire, light touch test	numb, tingling, painful etc.
15	NR	Pano, CT	2-point discrimination, light touch test, thermal test	altered sensation, anesthesia
16	NR	NR	NR	dysesthesia
17	7-20/3.75–4	NR	NR	disturbed nerve sensation
18	NR	NR	questionnaire, light touch test, pin-prick test	hypersensitivity, anesthesia
19	10-15/3.3–4	Pano	light touch test	dysesthesia, hypoesthesia, anesthesia
20	NR	NR	questionnaire	burning, painful etc.
21	NR	NR	questionnaire	burning, painful etc.
22	NR	Pano	NR	paresthesia
23	NR	Pano, tomograph	NR	hypoesthesia, anesthesia
24	NR	NR	questionnaire	NR
25	7-20/3.75–4	Pano	NR	paresthesia
26	NR	Pano	NR	NR

CT: computed tomography; DVT: digital volume tomograph; NR: not reported; Pano: panoramic radiograph.

*All implant length and diameter is measured in mm.

(C) Outcomes of altered sensation (4 time points and 3 items for each time point, see Table 4), including (1) the time point when the investigation was performed, (2) the number of the patients initially showing altered sensation, and (3) the number of total patients being investigated, and. The data were extracted separately for short-term, intermediate-term, long-term outcomes, and the outcomes reported at other time point. Therefore 12 items in total were recorded (Table 4).

**Table 4:** Outcomes of the included studies.

	Short-term			Intermediate-term			Long-term			Other time point of assessment		
ID	Time point of assessment	#Patients*		Time point of assessment	#Patients		Time point of assessment	#Patients		Time point of assessment	#Patients	
1	postsurgical	11	/40	NR	NR	NR	at their last recall visit (>5 year)	5	/40			
2	postoperative	10	/55	follow-up continued for >6 months	5	/55	follow-up continued for 2 years	4	/55			
3	NR	NR	NR	NR	NR	NR	3-year follow-up	0	/23			
4	NR	NR	NR	NR	NR	NR	NR	NR	NR	not explicitly specified	5	/13
5a	after implant placement	1	/15	NR	NR	NR	NR	NR	NR			
5b	after implant placement	3	/15	NR	NR	NR	NR	NR	NR			
6a	postoperative	16	/30	NR	NR	NR	NR	NR	NR			
6b	postoperative	2	/30	NR	NR	NR	NR	NR	NR			
7	postoperative	0	/20	NR	NR	NR	NR	NR	NR			
8	1 week after surgery	2	/1527	NR	NR	NR	NR	NR	NR			
9	NR	NR	NR	NR	NR	NR	NR	NR	NR	after implant placement (8–24 months)	19	/58
10	NR	NR	NR	NR	NR	NR	5.5 years after treatment	1	/17			
11	NR	NR	NR	NR	NR	NR	1 year after prosthesis placement	0	/60			
12	postoperative	2	/50	NR	NR	NR	NR	NR	NR			
13	NR	NR	NR	6 months after surgery	0	/6	1 year after surgery	0	/6			
14	NR	NR	NR	6 months after surgery	3	/75	1 year after surgery	1	/75			
15	within 1 week of surgery	8	/94	121 days after surgery	0	/94	NR	NR	NR			
16	postoperative	11	/50	NR	NR	NR	NR	NR	NR			
17	NR	NR	NR	NR	NR	NR	NR	NR	NR	during the entire study period; not-explicitly specified	1	/103
18	10 days after surgery	11	/103	NR	NR	NR	16 months after surgery	10	/102			
19	NR	NR	NR	NR	NR	NR	12–57 months after loading	0	/57			
20	1 week after surgery	24	/87	> 3 months duration	4	/87	> 1 year duration	1	/87			
21	1 week after surgery	50	/212	> 3 months duration	17	/212	> 1 year duration	4	/212			
22	after implant placement	19	/103	NR	NR	NR	1 year after surgery	1	/103			
23	NR	NR	NR	NR	NR	NR	2-year follow-up	7	/23			
24	NR	NR	NR	NR	NR	NR	NR	NR	NR	2 weeks after phase 1 surgery	14	/32
25	after implant placement	16	/91	NR	NR	NR	1 year after prosthesis placement	6	/91			
26	NR	NR	NR	NR	NR	NR	NR	NR	NR	during the 4–6 years observation after implant placement	0	/46

NR: not reported.

*The column ‘#patients’ denotes the number of patients who reported altered sensation / the number of patients who received implant placement.

It is noteworthy that some of the studies have reported the occurrence of altered sensation from more than one time point. For example, 6 studies have reported both short-term and long-term outcomes from the same cohort of patients [14,21,25–28]. Statistically, this overlap would increase the dependency between the results of different time point (see Discussion). However, because it is common for the clinical studies to examine patients’ symptoms for more than one time point, we here separately treated the outcomes from the same studies for different analyses. For example, the results from the study by Boven et al. ([25]) would be analysed for both short-term and long-term altered sensation.

### Assessment of Study Quality and Risk of Bias

Assessment of study quality and risk of bias was performed according to a 7-item customized assessment sheet, which was modified from the Newcastle-Ottawa Quality Scale (NOQS) [29], a scale specific for non-randomized cohort study. The customized scale assessed the representativeness of cases, the research methods and the outcomes, of an individual study (see Table 5 for details the items).

**Table 5:** Criteria of Assessment of Study Quality and Risk of Bias.

Original NOQS items		Modified items	Scoring	
			Yes	Unclear
Representativeness of the exposed cohort	(1)	Are the inclusion and exclusion criteria explicitly stated?	1	0
	(2)	Are the clinical features of the cohort explicitly stated?	1	0
Ascertainment of exposure	(3)	Are the surgical procedures explicitly stated?	1	0
Demonstration that outcome of interest was not present at start of study	(4)	Are the methods of assessment of altered sensation explicitly stated?	1	0
Assessment of outcome	(5)	Are the experiences of altered sensation explicitly defined?	1	0
Was follow-up long enough for outcomes to occur	(6)	Is the duration of follow-up explicitly stated?	1	0
Adequacy of follow up of cohorts	(7)	Is the general success/survival rate of implant placement explicitly stated?	1	0

### Data Synthesis

We calculated the pooled event rate using Metaprop [30], a Stata-based program specifically designed for binominal data. The confidence intervals (CIs) were calculated using an exact binominal approach. Conventionally, a proportion, such as the event rate of altered sensation, can be approximated by the normal distribution. However, when the proportions are close to the boundary (i.e., 0 and 1), the normal distribution may not be a good approximation of proportions [30]. We did not adopt the approximation approach, because in our meta-analysis, the event rates of altered sensation were 0 or 100% in several studies. Specifically, the analysis was performed based on Stata version 13.1, using the program *metaprop_one*[30]. We used the exact method (i.e., the option *cimethod(exact)*) to calculate the confidence intervals of each study. A random-effect analysis was performed, (i.e., with the option *logit*) so that a binomial distribution is used to model the within-study variability and the parameters were estimated using a maximum likelihood procedure. Two analyses were performed to estimate the incidence and the recovery rate of altered sensation:

1. Analysis 1: We investigated the incidence of altered sensation by calculating the pooled event rate of altered sensation, using a random-effect model. The analysis was performed respectively for (1) short-term (within 10 days after surgery) and (2) long-term (more than 1 year) outcomes. Regarding the intermediate-term outcome, we only descriptively examined the results from systematic review. We did not perform a meta-analysis on this time point, since the definition about ‘intermediate-term’, as we defined here (3 to 6 months after surgery) can be too ambiguous, and therefore would result in great between-study heterogeneity. Between-study heterogeneity was assessed using τ^2^ statistics. Here the value τ is variance in incidence between studies (here treated as a random effect)[31,32]. Statistical significance of τ^2^ was assessed using a random-effect model vs. fixed-effect model likelihood ratio (LR) test. We adopted a random-effect model effect because of the statistical heterogeneity between the studies (see Results) and the clinical heterogeneity due to experimental design and sampling [31].
2. Analysis 2: We investigated the recovery rate of initial altered sensation. *Recovery rate* was defined as the ratio of the patients whose altered sensation restored to normal sensation to those who had reported altered sensation postoperatively. *Recovery duration* was defined as the time when the clinical assessment was performed and normal sensation was recorded. The pooled recovery rate was calculated, respectively, for recovery duration ≦ 6 months and recovery duration = 1 year, using a random-effect model. These outcomes from were extracted based on the descriptions of the results or discussion in the original articles. For example, in one study (Study ID = 4), the authors have explicitly stated that ‘the maximum duration of these sensory disturbances was 6 weeks’ [33]. Therefore, we considered that all the patients who initially reported altered sensation (5 patients) returned to normal sensation within 6 months.

### Assessment of Publication Bias

The degree of publication bias was visualized with funnel plots, and the degree of asymmetry of the funnel plots were estimated based on the Egger’s regression test [34]. The analyses were performed using statistical software Comprehensive Meta-analysis 2 (Biostat, Engelwood, NJ).

## Results

### Results of Search and Study Selection

Our search yielded 196 articles, including 188 articles from computerized search and 8 articles from manual search (Fig 1). After screening, 170 articles were excluded for a variety of reasons (see S2 Table for the reasons of excluding the articles). 26 articles were finally included. Among these, 2 articles (Study ID = 5, 6) reported the findings about two different surgical procedures [35,36]. These articles were each considered as two studies, and thus 28 studies were included (Fig 1).

### Demographic, Clinical and Methodological Characteristics of the Included Studies

As shown in Tables 2 and 3, the included studies demonstrated a considerable degree of heterogeneity in the demographic, clinical and methodological aspects. The studies were predominantly an observational cohort study. Regarding the demographic and clinical aspects (Table 1), there were more female patients recruited in the studies, and the age was relatively elder, mostly 40–60 y/o. Patients’ mandibular ridges were moderate-to-severely atrophic, with inadequate bone height and/or poor bone quality. Reduction in the ridge-to-IAN canal distance was noted in several studies [35–37]. Augmentation procedures were performed in 5 studies [25,33,35,36,38]. Regarding the methodological aspects (Table 3), in most studies, an implant with a regular size (i.e., >10mm in length and 3.75-5mm in diameter) [39] was used. Panoramic radiography was frequently taken preoperatively, either alone (n = 9) or together with tomograph/computed tomography (n = 4). Regarding the assessment of altered sensation, questionnaires were used in 6 studies, and sensory testing was performed in 8 studies, among which a light touch test was the most frequently performed (n = 6). Paresthesia was the most recognized experience of altered sensation (n = 10), followed by pain (n = 6), anesthesia (n = 5), hypoesthesia (n = 4), and dysesthesia (n = 2).

### Study Quality and Risk of Bias

Based on the results of study quality and risk of bias assessment, we found 3 studies with a score between 0 and 2 (Study ID = 2, 7, 12), 4 studies with a score 3(Study ID = 13, 16, 20, 26), and 21 studies with a score between 4 and 7 (for details see S3 Table). The average score of the assessment was 4.1±1.4 (±standard deviation), based on our customized scale (Table 5). In general, the study quality and risk of bias was moderate, with a substantial degree of variability across the studies. Regarding the representativeness of sampling, 50% of the studies have provided clear information about both the inclusive and exclusive criteria of patient selection. 64% of the studies have clearly stated the source of patient sampling (e.g., from multiple centers or a single hospital). 79% of the studies have clearly stated the surgical methods for implant placement. Regarding outcome assessment, only 36% of the studies have clearly defined the experience of altered sensation before assessment, and 54% of the studies have clearly stated the methods for assessment. Finally, 68% and 64% of the studies have clearly stated the duration of follow-up and success/survival rate of surgery, respectively (for detailed results see S3 Table).

### Analysis 1: Estimating the Short-term and Long-term Incidence of Altered Sensation

The meta-analyses included 16 studies for estimating the short-term incidence (Study ID = 1, 2, 5a, 5b, 6a, 6b, 7, 8. 12, 15, 16, 18, 20, 21, 22, 25), 6 studies for the intermediate-term incidence (Study ID = 2, 13, 14, 15, 20, 21) and 14 studies for the long-term incidence (Study ID = 1, 2, 3, 10, 11, 13, 14, 18, 19, 20, 21, 22, 23, 25) (Table 4). The results of the intermediate-term outcome were discussed descriptively. Additionally, in 5 studies (Study ID = 4, 9, 17, 24, 26) the time point of altered sensation assessment is not confined to the short-term, intermediate-term or long-term category. For example, one study (Study ID = 4) reported altered sensation in 5 out of 13 patients; however the time-point when the assessment of altered sensation was performed is not explicitly stated in the article [33]. The outcomes from these studies were categorized as ‘Other time point of assessment’ in Table 4. These outcomes would be discussed descriptively and not be included in the meta-analysis.

**Fig 2:**
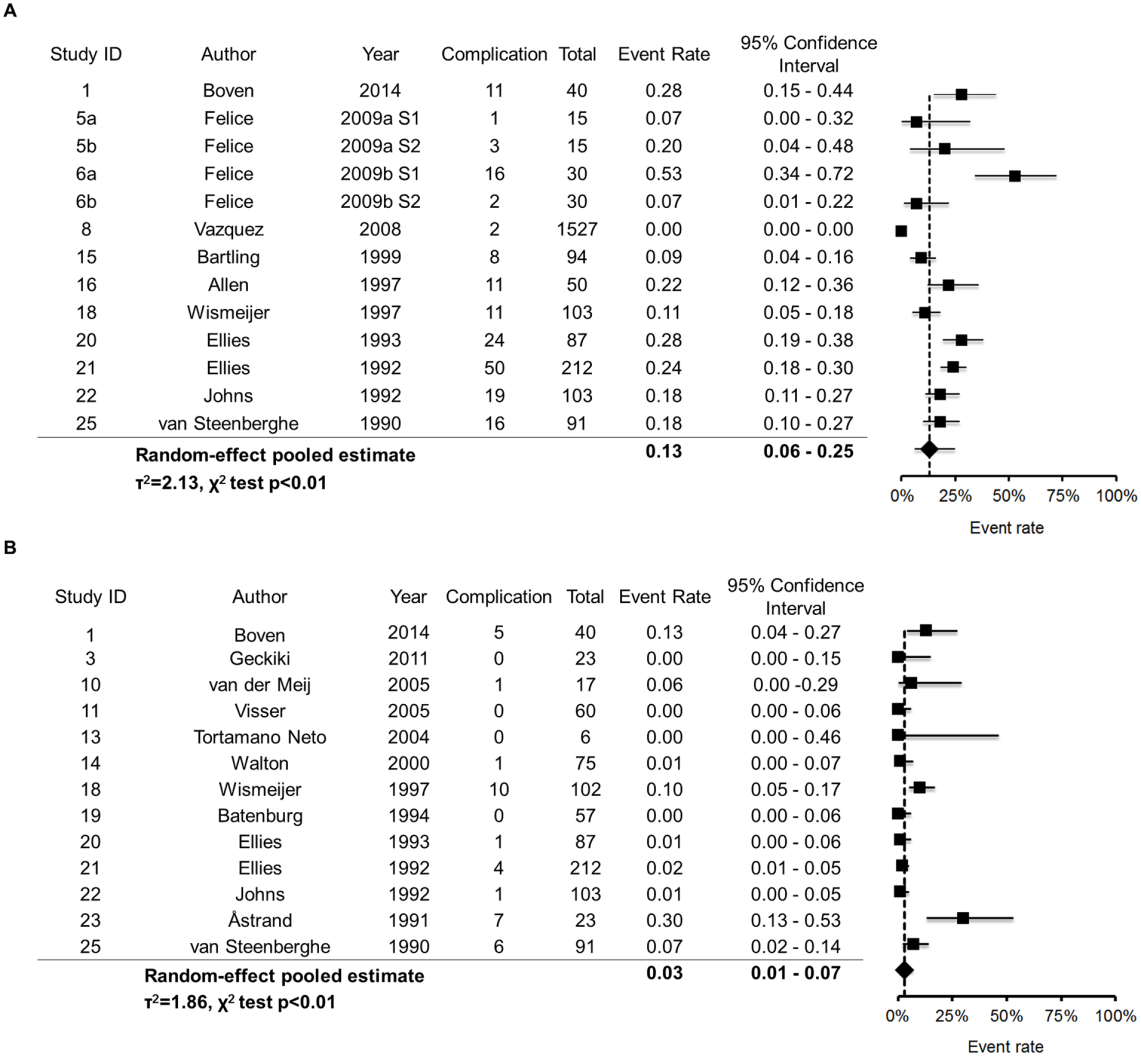
Forest plots of the incidence of altered sensation. **(A)** The short-term (within 10 days after surgery) incidence. **(B)** The long-term (1 year after surgery) incidence.

It should be noted that the results of study quality and risk of bias showed a higher level of variance, which may influence the pooled estimation of incidence. Therefore, we conducted data synthesis after excluding the studies with a lower score (0–2) of the study quality and risk of bias assessment. After the adjustment, 3 studies (Study ID = 2, 7, 12) were excluded for the analysis of short-term incidence and 1 study (Study ID = 2) was excluded for the analysis of long-term incidence. The short-term incidence was 13% (95% CI, 6%-25%, Fig 2A) and the long-term incidence was 3% (95% CI, 1%-7%, Fig 2B). A significant degree of between-study heterogeneity was found for both the short-term analysis (LR test: χ^2^_(1)_ = 348.2, p<0.01, τ^2^ = 2.13) and the long-term analysis (LR test: χ^2^_(1)_ = 23.6, p<0.01, τ^2^ = 1.86). Without the adjustment for study quality and risk of bias, the short-term incidence was 11% (95% CI, 5%- 21%; LR test: χ^2^_(1)_ = 350.9, p<0.01, τ^2^ = 2.07) and the long-term incidence was 3% (95% CI, 1%-7%; LR test: χ^2^_(1)_ = 22.3, p<0.01, τ^2^ = 1.60).

### Analysis 2: Estimating the Recovery Rate of Altered Sensation

Fifteen studies that provided the information about recovery rate were included in the analysis (for detailed results see Table 6). Note that the studies included in Analysis 2 (Table 6) may not be identical to the studies included in Analysis 1 (Table 4). For example, one study (Study ID = 16) reported the patients with postoperative altered sensation, but it did not explicitly state the duration that the patients recovered from altered sensation [40]. These studies were excluded in Analysis 2. For another example, one study (Study ID = 4) reported the duration of recovery, but it did not explicitly state the time point of assessment [33]. These studies (Study ID = 4, 9, 18) would be included in Analysis 2 but not in Analysis 1.

**Table 6:** Recovery rate and recovery duration.

ID	Recovery duration (month)	#Patients recovered to normal sensation	#Patients reported altered sensation	Source of the outcomes	Recovery rate
1	60	6	11	p.629: ‘Eleven patients reported postsurgical sensory disturbances of the mental nerve (objectively and subjectively), five of whom still had a sensory disturbance in the chin region at their last recall visit, but the size of this region had diminished over time.’	55%
2	1	5	10	p.e406: ‘Pain duration of the patients ranged from 1 month to 4 years. Neuropathic pain has continued only for 1 month in 50%, 6 months in 10%, and 2 years in 10% of the patients after the operation.’	50%
2	6	6	10	Same as above	60%
2	24	7	10	Same as above	70%
4	1.5	5	5	pp.556-557: ‘None of these patients complained of permanent sensory disturbances. The maximum duration of these sensory disturbances was 6 weeks.’	100%
5a	0.1	1	1	p.278: ‘No permanent paraesthesia of the alveolar inferior nerve occurred, the longest lasting 3 days.’	100%
5b	0.1	3	3	Same as above	100%
8	1.5	2	2	p.83: ‘The nerve-altered sensation lasted for 6 weeks and disappeared without treatment.’	100%
9	3	11	19	p.272:‘The duration of this postsurgical neurosensory disturbance after the implant surgery was less than 3 months in 58% (n = 11)’	58%
12	1	2	2	p.493: ‘The altered sensation in the lower lip and the skin area of the chin was present for about three weeks in patient one and four weeks in patient two. After that time, both patients reported normal sensation.’	100%
14	6	15	18	p.466: ‘Although approximately 24% of all subjects reported sensation changes at 2 weeks after stage 1 surgery, only approximately 4% still reported sensory changes at 6 months after initial surgery, dropping to about 1%, 12 months later.’	83%
14	12	17	18	Same as above	94%
15	4	8	8	p.1410: ‘All patients had returned to normal nerve function by 121 days after implant placement.’	100%
18	16	5	11	Table 4^1^	45%
20	1	11	20	Table 2 and p.676: ‘When transient changes in sensation were experienced, 90% of patients reported that the symptoms had resolved by 6 months (Table 2).’^2^	55%
20	6	18	20	Same as above	90%
20	12	19	20	Same as above	95%
21	1	24	51	Table V and p.666: ‘Transient alterations in sensation had resolved by 6 months in more than 80% of patients; however, four patients reported symptoms that resolved after 2 to 3 years (Table V).’	47%
21	6	42	51	Same as above	82%
21	12	47	51	Same as above	92%
22	12	18	19	p.518: ‘One of the remaining patients still had signs of paresthesia after 1 year of function.’	95%
25	12	10	16	p.277: ‘One year after prosthesis placement, the reported complications were dominated by patients with prosthetic difficulties, such as loosening of gold screws and fractures of the resin material (n = 10). Furthermore, six patients still complained of remaining paresthesia.’	63%

^1^According to Table 4, 11 patients reported altered sensation on at least on side immediately after implant treatment. Among the 11 patients (No. 47, 49, 59, 69, 73, 77, 80, 85, 86, 87, 98), 5 patients (No. 47, 49, 73, 80, 85) have recovered to normal sensation within 16 months after treatment. The exact duration of altered sensation was not available.

^2^Only the patients with transient altered sensation (n = 20) were considered here. The patients with persistent changes were excluded because the exact duration of altered sensation was not available from the article.

**Fig 3:**
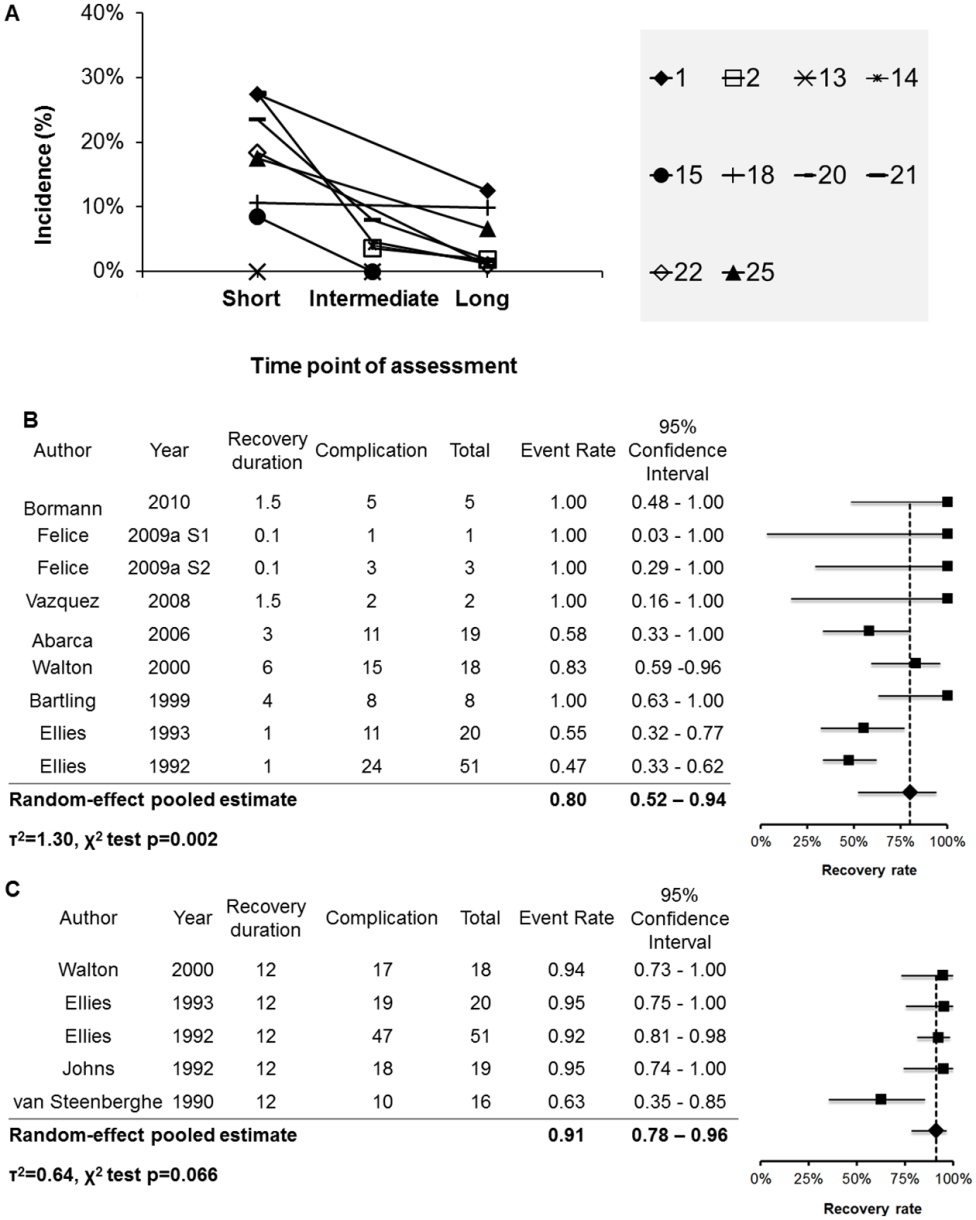
Recovery rate of altered sensation. **(A)** The incidence of altered sensation through the duration of follow up. All the studies have assessed the occurrence of altered sensation for at least two different time points, according to the outcomes presented in Table 4. Study IDs of the included studies are shown in the legend. **(B)** The forest plot for the recovery rate, when recovery duration ≦ 6 months. **(C)** The forest plot for the recovery rate, when recovery duration = 1 year.

As shown in Fig 3A, the studies have shown a declined incidence of altered sensation during the follow-up sessions. After the excluding the studies with a lower score (0–2) from the study quality and risk of bias assessment (Study ID = 2, 12), 9 studies (Study ID: 4, 5a, 5b, 8, 9, 14, 15, 20, 21) reported the recovery rate within 6 months after surgery with the pooled recovery rate 80% (95% CI, 52%-94%, Fig 3B). A significant degree of between-study heterogeneity (LR test: χ^2^_(1)_ = 7.9, p<0.01, τ^2^ = 1.30) was found. Five studies (Study ID: 14, 20, 21, 22, 25) reported the recovery rate 1 year after surgery (Table 6), with the pooled recovery rate 91% (95% CI, 78%-96%, Fig 3C). The between-study heterogeneity was not statistically significant (LR test: χ^2^_(1)_ = 2.26, p = 0.07, τ^2^ = 0.64). Without the adjustment for study quality and risk of bias, the recovery rate within 6 months was 77% (95% CI, 53%- 91%; LR test: χ^2^_(1)_ = 7.0, p<0.01, τ^2^ = 1.05). The recovery rate for 1 year did not differ before and after adjustment, because no study was excluded in this analysis.

### Publication Bias

Regarding the analysis of short-term and long-term incidence, the Egger’s regression test showed significant asymmetry (P = 0.03 and P = 0.03, respectively) of the funnel plot. Regarding the analysis of intermediate-term, Egger’s regression test did not show significant asymmetry (P = 0.13) of the funnel plot (see S1 Fig for the funnel plots).

## Discussion

### Summary of Major Findings

We here reported the incidence of altered sensation after mandibular implant surgery based on a meta-analysis of 26 articles (28 studies) between 1990 and 2016. We concluded three major findings:

1. The short-term (within 10 days after surgery) and long-term (1 year after implant placement) incidence was 13% (95% CI, 6%-25%) and 3% (95% CI, 1%-7%), respectively (Fig 2).
2. All studies have shown a declined incidence during the follow-up session (Fig 3A). When altered sensation was found immediately after surgery, within 6 months after surgery, 80% (95% CI, 52%-94%) of the patients would return to normal sensation (Fig 3B). One year after surgery, 91% (95% CI, 78%-96%) of them would return to normal sensation (Fig 3C).

### Incidence of Altered Sensation and the Recovery Rate after Mandibular Implant Placement

Estimates of the incidence of altered sensation varied dramatically across previous studies. A frequently cited number is 8.5% according to Bartling and co-workers, based on the results of subjective assessment and sensory testing of 94 patients [41]. An earlier retrospective research by Ellies reported an incidence 37%, based on the questionnaires from 226 multi-center patients [14]. Another earlier systematic review by Berglundh and co-workers estimated that 41% of the 159 reviewed studies have reported an occurrence of persistent (lasting more than 1 year) sensory disturbance [10]. In contrast, according to Vazquez and co-workers, based on a larger cohort of patients (n = 1527), the incidence was very low (0.13%) [13]. Therefore, most researchers would estimate the incidence with a great degree of variability, such as 0–15% [8] or 0–40% [15]. The huge variation may be associated with the heterogeneity in research methods. While some studies only focused on persistent changes (e.g., disturbances lasting for more than 1 year), others focused on transient ones (e.g., disturbances occurring immediately after surgery). We here estimated the short-term and long-term incidence of altered sensation. As expected, the incidence decreased through the three time points. Our meta-analysis results are consistent with the neurophysiological model of nerve injury, which classified nerve injury into three major classes: neuropraxia, axonotmesis and neurotmesis [19,20]. A transient and reversible injury without damaging the nerve proper and nerve sheath i.e., neuropraxia or axonotmesis, would spontaneously return to normal sensation within 2 to 4 months, as shown in most of the cases of altered sensation after mandibular implant surgery [42,43].

Furthermore, we found the recovery rate was high (80%) for the first 6 months after implant placement. One year after implant placement, an even higher proportion of patients (91%) may return to normal sensation (Fig 3B). The findings suggested that, even though the short-term assessment showed a considerably high incidence (13%) of altered sensation, most of the cases would be resolved within 1 year.

### Effects of Biological Risk Factors

The elderly patients usually have a severely atrophic ridge, with a short distance between the ridge and the IAN [16,17]. The influences of patients’ age and the degree of ridge atrophy on the incidence of altered sensation have not been fully assessed. The results from the review suggested that short ridge-to-IAN canal distance may not influence the short-term incidence of altered sensation. It should be noted that in 4 of the short-RCD studies (Study ID = 5a, 5b, 6a, 6b), either augmentation procedures were performed or a short implant (5mm or 7mm) was used, in order to compensate the inadequate bone height [35,36]. The finding suggested that ridge atrophy (RCD < 10mm) may not significantly increase the risk of altered sensation, given that augmentation procedures are performed or a shorter implant is used. The finding highlighted the importance of pre-surgery treatment planning. The anatomical relation between the implant and the IAN, especially the mental nerve branch, plays a critical role in the occurrence of altered sensation [42,43]. Imaging techniques, such as the computed tomography would be valuable tools for precisely evaluating the spatial relation between bone and nerve structures [44]. The problems are particularly critical to the elderly patients of implant surgery, because both the degree of ridge atrophy and bone quality is related with aging [45]. Our analysis did not reveal a significant association between age and the incidence of altered sensation. This negative finding implies that age per se may not have a direct effect on altered sensation. Instead, its effect may be associated with multiple factors, such as ridge morphology and bone quality/quantity. Interaction between these factors would require further investigation.

### Limitations and Further Considerations

Due to the heterogeneity in study design, patient sampling and methods of assessment across the studies, conclusions from the current review and meta-analysis should be carefully interpreted with the following considerations:

1. For the biological risk factors, we here only evaluated the effect of age and ridge-to-IAN canal distance, since these factors can be precisely quantified. However, we did not investigate the overall physical and behavioral conditions, such as diabetes and smoking, which are known associated with implant outcomes [46,47]. For example, diabetes is associated with altered pain sensitivity in the elderly patients [48]. As shown in the assessment of risk of bias, few studies have systematically evaluated these risk factors during patient sampling. The effect of these factors would require further investigation.
2. Regarding the methods of assessment, we here did not differentiate the specific experiences of altered sensation. Clinically, dysesthesia or pain may indicate the development of neuropathy or a significant influence on patients’ quality of life [49,50]. However, because most studies did not clearly state how these experiences were defined to the patients, we here pooled all the symptoms under an umbrella term ‘altered sensation’. In addition, very few studies have investigated the quantity of altered sensation, for example, using a psychophysical scale (e.g., the numerical rating scale). Therefore, we cannot evaluate the change about the intensity of altered sensation through a period of time.
3. Regarding the method of data synthesis, it should be noted that the estimates of short-term and long-term altered sensation were from some overlapped studies– 6 studies have reported both short-term and long-term results [14,21,25–28]. Although this is commonly seen in the clinical studies about post-surgical results, in which the authors would usually examine patients’ symptoms for more than one time point. The lack of independence may lead to an underestimation of standard errors and hence confidence intervals.
4. It should be noted that, in terms of surgical procedure, both drilling and implant placement may induce IAN injury. However, the included studies did not distinguish between the two conditions. It has remained unknown whether drilling or placement is the dominant factor to IAN injury. Apart from the physical conditions, altered sensation is both a sensory and an emotional experience [51]. Very few studies have reported the emotional aspects about altered sensation, and the psychosocial profiles about patients with altered sensation were mostly under-researched (except for [52,53], for example). Importantly, when patients perceive altered sensation after surgery, the increased dissatisfaction and negative emotion may results in liability claims. The dissatisfaction would be greater if the risk of altered sensation is not notified before surgery. The association between the affective-motivational factors and the medico-legal claims have not been fully investigated.

### Clinical Implications

Based on our findings, we suggested the following aspects about improving the quality of dental implant therapy.

1. On dentist-patient communication about the risk of altered sensation: our findings showed that, for the implant surgery with conventional procedures, the risk to have altered sensation immediately after surgery is substantial (13%). Nevertheless, most of these initial symptoms would spontaneously return to normal. Our findings highlighted the importance of early assessment[11]. Importantly, when a patient reports altered sensation, regular assessment for 6 months would be critical to trace the changes of altered sensation.
2. On the timing of assessment: our findings suggested that an assessment during 3 to 6 months postoperatively would be valuable for subsequent management of altered sensation. Our finding echoed the proposal that if the symptom does not reduce by the time of 12 weeks, further intervention (e.g., neurosurgical referral) may be considered [42,43]. We suggested that follow-up assessment should be scheduled no shorter than 6 months, since most patients with initial altered sensation may return to normal at that stage.

## Supporting Information

S1 Fig Publication Bias. (TIF)

S1 Table PRISMA-P Checklist. (DOCX)

S2 Table Reasons for Article Exclusion. (DOCX)

S3 Table Results of Assessment of Study Quality and Risk of Bias. (DOCX)

## References

[1] Al-Sabbagh M, Okeson JP, Bertoli E, Medynski DC, Khalaf MW (2015) Persistent pain and neurosensory disturbance after dental implant surgery: prevention and treatment. Dent Clin North Am 59: 143–156. 10.1016/j.cden.2014.08.005 25434563

[2] Chaushu G, Taicher S, Halamish-Shani T, Givol N (2002) Medicolegal aspects of altered sensation following implant placement in the mandible. Int J Oral Maxillofac Implants 17: 413–415. 12074458

[3] Delcanho R, Moncada E (2014) Persistent pain after dental implant placement: a case of implant-related nerve injury. J Am Dent Assoc 145: 1268–1271. 25429041 10.14219/jada.2014.210

[4] Al-Sabbagh M, Okeson JP, Khalaf MW, Bhavsar I (2015) Persistent pain and neurosensory disturbance after dental implant surgery: pathophysiology, etiology, and diagnosis. Dent Clin North Am 59: 131–142. 10.1016/j.cden.2014.08.004 25434562

[5] Figgener L, Kleinheinz J (2004) Implant dentistry at the focus of liability lawsuits. Int J Oral Maxillofac Implants 19: 382–386. 15214222

[6] Albrektsson T, Zarb G, Worthington P, Eriksson AR (1986) The long-term efficacy of currently used dental implants: a review and proposed criteria of success. Int J Oral Maxillofac Implants 1: 11–25. 3527955

[7] Papaspyridakos P, Chen CJ, Singh M, Weber HP, Gallucci GO (2012) Success criteria in implant dentistry: a systematic review. J Dent Res 91: 242–248. 10.1177/0022034511431252 22157097

[8] Bagheri SC, Meyer RA (2014) When to refer a patient with a nerve injury to a specialist. J Am Dent Assoc 145: 859–861. 10.14219/jada.2014.45 25082936

[9] Coulthard P, Kushnerev E, Yates JM, Walsh T, Patel N, Bailey E, et al (2014) Interventions for iatrogenic inferior alveolar and lingual nerve injury. Cochrane Database Syst Rev 4: CD005293 10.1002/14651858.CD005293.pub2 24740534 PMC10794896

[10] Berglundh T, Persson L, Klinge B (2002) A systematic review of the incidence of biological and technical complications in implant dentistry reported in prospective longitudinal studies of at least 5 years. J Clin Periodontol 29 Suppl 3: 197–212; discussion 232–193. 12787220 10.1034/j.1600-051x.29.s3.12.x

[11] Shavit I, Juodzbalys G (2014) Inferior alveolar nerve injuries following implant placement—importance of early diagnosis and treatment: a systematic review. J Oral Maxillofac Res 5: e2. 10.5037/jomr.2014.5402 PMC4306320 25635209

[12] Vetromilla BM, Moura LB, Sonego CL, Torriani MA, Chagas OL Jr. (2014) Complications associated with inferior alveolar nerve repositioning for dental implant placement: a systematic review. Int J Oral Maxillofac Surg 43: 1360–1366. 10.1016/j.ijom.2014.07.010 25128261

[13] Vazquez L, Saulacic N, Belser U, Bernard JP (2008) Efficacy of panoramic radiographs in the preoperative planning of posterior mandibular implants: a prospective clinical study of 1527 consecutively treated patients. Clin Oral Implants Res 19: 81–85. 17956572 10.1111/j.1600-0501.2007.01402.x

[14] Ellies LG (1992) Altered sensation following mandibular implant surgery: a retrospective study. J Prosthet Dent 68: 664–671. 1403947 10.1016/0022-3913(92)90384-m

[15] Juodzbalys G, Kubilius M (2013) Clinical and radiological classification of the jawbone anatomy in endosseous dental implant treatment. J Oral Maxillofac Res 4: e2. 10.5037/jomr.2013.4202 PMC3886111 24422030

[16] Raghoebar GM, Meijer HJ, Stellingsma K, Vissink A (2011) Addressing the atrophied mandible: a proposal for a treatment approach involving endosseous implants. Int J Oral Maxillofac Implants 26: 607–617. 21691609

[17] Stellingsma C, Vissink A, Meijer HJ, Kuiper C, Raghoebar GM (2004) Implantology and the severely resorbed edentulous mandible. Crit Rev Oral Biol Med 15: 240–248. 15284188 10.1177/154411130401500406

[18] Moher D, Shamseer L, Clarke M, Ghersi D, Liberati A, Petticrew M, et al (2015) Preferred reporting items for systematic review and meta-analysis protocols (PRISMA-P) 2015 statement. Syst Rev 4: 1 10.1186/2046-4053-4-1 25554246 PMC4320440

[19] Seddon HJ (1942) A Classification of Nerve Injuries. Br Med J 2: 237–239. 20784403 10.1136/bmj.2.4260.237 PMC2164137

[20] Akal UK, Sayan NB, Aydogan S, Yaman Z (2000) Evaluation of the neurosensory deficiencies of oral and maxillofacial region following surgery. Int J Oral Maxillofac Surg 29: 331–336. 11071233

[21] Ellies LG, Hawker PB (1993) The prevalence of altered sensation associated with implant surgery. Int J Oral Maxillofac Implants 8: 674–679. 8181830

[22] Poort LJ, van Neck JW, van der Wal KG (2009) Sensory testing of inferior alveolar nerve injuries: a review of methods used in prospective studies. J Oral Maxillofac Surg 67: 292–300. 10.1016/j.joms.2008.06.076 19138602

[23] Cawood JI, Howell RA (1988) A classification of the edentulous jaws. Int J Oral Maxillofac Surg 17: 232–236. 3139793 10.1016/s0901-5027(88)80047-x

[24] Lekholm U, Zarb G (1985) Patient selection and preparation In: Branemark P-I, Zarb G, Albrektsson T, editors. Tissue-integrated Prostheses: Osseointegration in Clinical Dentistry. Chicago: Quintessence pp. 199–209.

[25] Boven GC, Meijer HJ, Vissink A, Raghoebar GM (2014) Reconstruction of the extremely atrophied mandible with iliac crest onlay grafts followed by two endosteal implants: a retrospective study with long-term follow-up. Int J Oral Maxillofac Surg 43: 626–632. 10.1016/j.ijom.2013.11.003 24411276

[26] Johns RB, Jemt T, Heath MR, Hutton JE, McKenna S, McNamara DC, et al (1992) A multicenter study of overdentures supported by Branemark implants. Int J Oral Maxillofac Implants 7: 513–522. 1299648

[27] van Steenberghe D, Lekholm U, Bolender C, Folmer T, Henry P, Herrmann I, et al (1990) Applicability of osseointegrated oral implants in the rehabilitation of partial edentulism: a prospective multicenter study on 558 fixtures. Int J Oral Maxillofac Implants 5: 272–281. 2098330

[28] Wismeijer D, van Waas MA, Vermeeren JI, Kalk W (1997) Patients' perception of sensory disturbances of the mental nerve before and after implant surgery: a prospective study of 110 patients. Br J Oral Maxillofac Surg 35: 254–259. 9291263 10.1016/s0266-4356(97)90043-7

[29] Wells G, Shea B, O’connell D, Peterson J, Welch V, Losos M, et al. (2000) The Newcastle-Ottawa Scale (NOS) for assessing the quality of nonrandomised studies in meta-analyses.

[30] Nyaga VN, Arbyn M, Aerts M (2014) Metaprop: a Stata command to perform meta-analysis of binomial data. Arch Public Health 72: 39 10.1186/2049-3258-72-39 25810908 PMC4373114

[31] Higgins JPT, Green S (2011) Cochrane Handbook for Systematic Reviews of Interventions. The Cochrane Collaboration.

[32] Higgins JP (2008) Commentary: Heterogeneity in meta-analysis should be expected and appropriately quantified. Int J Epidemiol 37: 1158–1160. 10.1093/ije/dyn204 18832388

[33] Bormann KH, Suarez-Cunqueiro MM, von See C, Kokemuller H, Schumann P, Gellrich NC (2010) Sandwich osteotomy for vertical and transversal augmentation of the posterior mandible. Int J Oral Maxillofac Surg 39: 554–560. 10.1016/j.ijom.2010.03.002 20435437

[34] Egger M, Davey Smith G, Schneider M, Minder C (1997) Bias in meta-analysis detected by a simple, graphical test. BMJ 315: 629–634. 9310563 10.1136/bmj.315.7109.629 PMC2127453

[35] Felice P, Cannizzaro G, Checchi V, Marchetti C, Pellegrino G, Censi P, et al (2009) Vertical bone augmentation versus 7-mm-long implants in posterior atrophic mandibles. Results of a randomised controlled clinical trial of up to 4 months after loading. Eur J Oral Implantol 2: 7–20. 20467615

[36] Felice P, Checchi V, Pistilli R, Scarano A, Pellegrino G, Esposito M (2009) Bone augmentation versus 5-mm dental implants in posterior atrophic jaws. Four-month post-loading results from a randomised controlled clinical trial. Eur J Oral Implantol 2: 267–281. 20467603

[37] Burstein J, Mastin C, Le B (2008) Avoiding injury to the inferior alveolar nerve by routine use of intraoperative radiographs during implant placement. J Oral Implantol 34: 34–38. 10.1563/1548-1336(2008)34[34:AITTIA]2.0.CO;2 18390241

[38] van der Meij EH, Blankestijn J, Berns RM, Bun RJ, Jovanovic A, et al (2005) The combined use of two endosteal implants and iliac crest onlay grafts in the severely atrophic mandible by a modified surgical approach. Int J Oral Maxillofac Surg 34: 152–157. 15695044 10.1016/j.ijom.2004.05.007

[39] Mijiritsky E, Mazor Z, Lorean A, Levin L (2013) Implant diameter and length influence on survival: interim results during the first 2 years of function of implants by a single manufacturer. Implant Dent 22: 394–398. 23811719 10.1097/ID.0b013e31829afac0

[40] Allen PF, McMillan AS, Smith DG (1997) Complications and maintenance requirements of implant-supported prostheses provided in a UK dental hospital. Br Dent J 182: 298–302. 9154708 10.1038/sj.bdj.4809371

[41] Bartling R, Freeman K, Kraut RA (1999) The incidence of altered sensation of the mental nerve after mandibular implant placement. J Oral Maxillofac Surg 57: 1408–1412. 10596660 10.1016/s0278-2391(99)90720-6

[42] Hegedus F, Diecidue RJ (2006) Trigeminal nerve injuries after mandibular implant placement—practical knowledge for clinicians. Int J Oral Maxillofac Implants 21: 111–116. 16519189

[43] Renton T (2010) Prevention of iatrogenic inferior alveolar nerve injuries in relation to dental procedures. Dent Update 37: 350–352, 354–356, 358–360 passim. 20929149 10.12968/denu.2010.37.6.350

[44] Bornstein MM, Al-Nawas B, Kuchler U, Tahmaseb A (2014) Consensus statements and recommended clinical procedures regarding contemporary surgical and radiographic techniques in implant dentistry. Int J Oral Maxillofac Implants 29 Suppl: 78–82. 10.11607/jomi.2013.g1 24660191

[45] Porter JA, von Fraunhofer JA (2005) Success or failure of dental implants? A literature review with treatment considerations. Gen Dent 53: 423–432; quiz 433, 446. 16366052

[46] Chrcanovic BR, Albrektsson T, Wennerberg A (2014) Diabetes and oral implant failure: a systematic review. J Dent Res 93: 859–867. 10.1177/0022034514538820 24928096 PMC4541101

[47] Chrcanovic BR, Albrektsson T, Wennerberg A (2014) Reasons for failures of oral implants. J Oral Rehabil 41: 443–476. 10.1111/joor.12157 24612346

[48] Pabbidi RM, Yu SQ, Peng S, Khardori R, Pauza ME, Premkumar LS (2008) Influence of TRPV1 on diabetes-induced alterations in thermal pain sensitivity. Mol Pain 4: 9. 18312687 10.1186/1744-8069-4-9 PMC2275252

[49] Klit H, Hansen AP, Marcussen NS, Finnerup NB, Jensen TS (2014) Early evoked pain or dysesthesia is a predictor of central poststroke pain. Pain 155: 2699–2706. 10.1016/j.pain.2014.09.037 25284071

[50] Smith JG, Elias LA, Yilmaz Z, Barker S, Shah K, Shah S (2013) The psychosocial and affective burden of posttraumatic neuropathy following injuries to the trigeminal nerve. J Orofac Pain 27: 293–303. 10.11607/jop.1056 24171179

[51] IASP (1994) Part III: Pain Terms, A Current List with Definitions and Notes on Usage In: Merskey H, Bogduk N, editors. Classification of Chronic Pain. 2nd ed Seattle: IASP Press.

[52] Abarca M, van Steenberghe D, Malevez C, De Ridder J, Jacobs R (2006) Neurosensory disturbances after immediate loading of implants in the anterior mandible: an initial questionnaire approach followed by a psychophysical assessment. Clin Oral Investig 10: 269–277. 16937108 10.1007/s00784-006-0065-0 PMC1705496

[53] Kiyak HA, Beach BH, Worthington P, Taylor T, Bolender C, Evans J (1990) Psychological impact of osseointegrated dental implants. Int J Oral Maxillofac Implants 5: 61–69. 2202671

[54] Kutuk N, Demirbas AE, Gonen ZB, Topan C, Kilic E, Etoz OA, et al (2013) Anterior mandibular zone safe for implants. J Craniofac Surg 24: e405–408. 10.1097/SCS.0b013e318292c7d5 23851883

[55] Geckili O, Bilhan H, Mumcu E (2011) Clinical and radiographic evaluation of three-implant-retained mandibular overdentures: a 3-year retrospective study. Quintessence Int 42: 721–728. 21909496

[56] Visser A, Raghoebar GM, Meijer HJ, Batenburg RH, Vissink A (2005) Mandibular overdentures supported by two or four endosseous implants. A 5-year prospective study. Clin Oral Implants Res 16: 19–25. 15642027 10.1111/j.1600-0501.2004.01085.x

[57] Frei C, Buser D, Dula K (2004) Study on the necessity for cross-section imaging of the posterior mandible for treatment planning of standard cases in implant dentistry. Clin Oral Implants Res 15: 490–497. 15248885 10.1111/j.1600-0501.2004.01032.x

[58] Tortamano Neto P, Camargo LO (2004) Prospective clinical evaluation of dental implants with sand-blasted, large-grit, acid-etched surfaces loaded 6 weeks after surgery. Quintessence Int 35: 717–722. 15470995

[59] Walton JN (2000) Altered sensation associated with implants in the anterior mandible: a prospective study. J Prosthet Dent 83: 443–449. 10756294 10.1016/s0022-3913(00)70039-4

[60] Friberg B, Grondahl K, Lekholm U (1992) A new self-tapping Branemark implant: clinical and radiographic evaluation. Int J Oral Maxillofac Implants 7: 80–85. 1398828

[61] Batenburg RH, van Oort RP, Reintsema H, Brouwer TJ, Raghoebar GM, Boering G (1994) Overdentures supported by two IMZ implants in the lower jaw. A retrospective study of peri-implant tissues. Clin Oral Implants Res 5: 207–212. 7640334 10.1034/j.1600-0501.1994.050403.x

[62] Astrand P, Borg K, Gunne J, Olsson M (1991) Combination of natural teeth and osseointegrated implants as prosthesis abutments: a 2-year longitudinal study. Int J Oral Maxillofac Implants 6: 305–312. 1813398

[63] Zarb GA, Schmitt A (1990) The longitudinal clinical effectiveness of osseointegrated dental implants: the Toronto study. Part III: Problems and complications encountered. J Prosthet Dent 64: 185–194. 2202818 10.1016/0022-3913(90)90177-e

