# Supplementary figures and images for: Systematic Review and Meta-Analysis on Incidence of Altered Sensation of Mandibular Implant Surgery
Source: PLoS One. 2016 Apr 21;11(4):e0154082. doi: 10.1371/journal.pone.0154082 (PMC4839635; doi:10.1371/journal.pone.0154082)

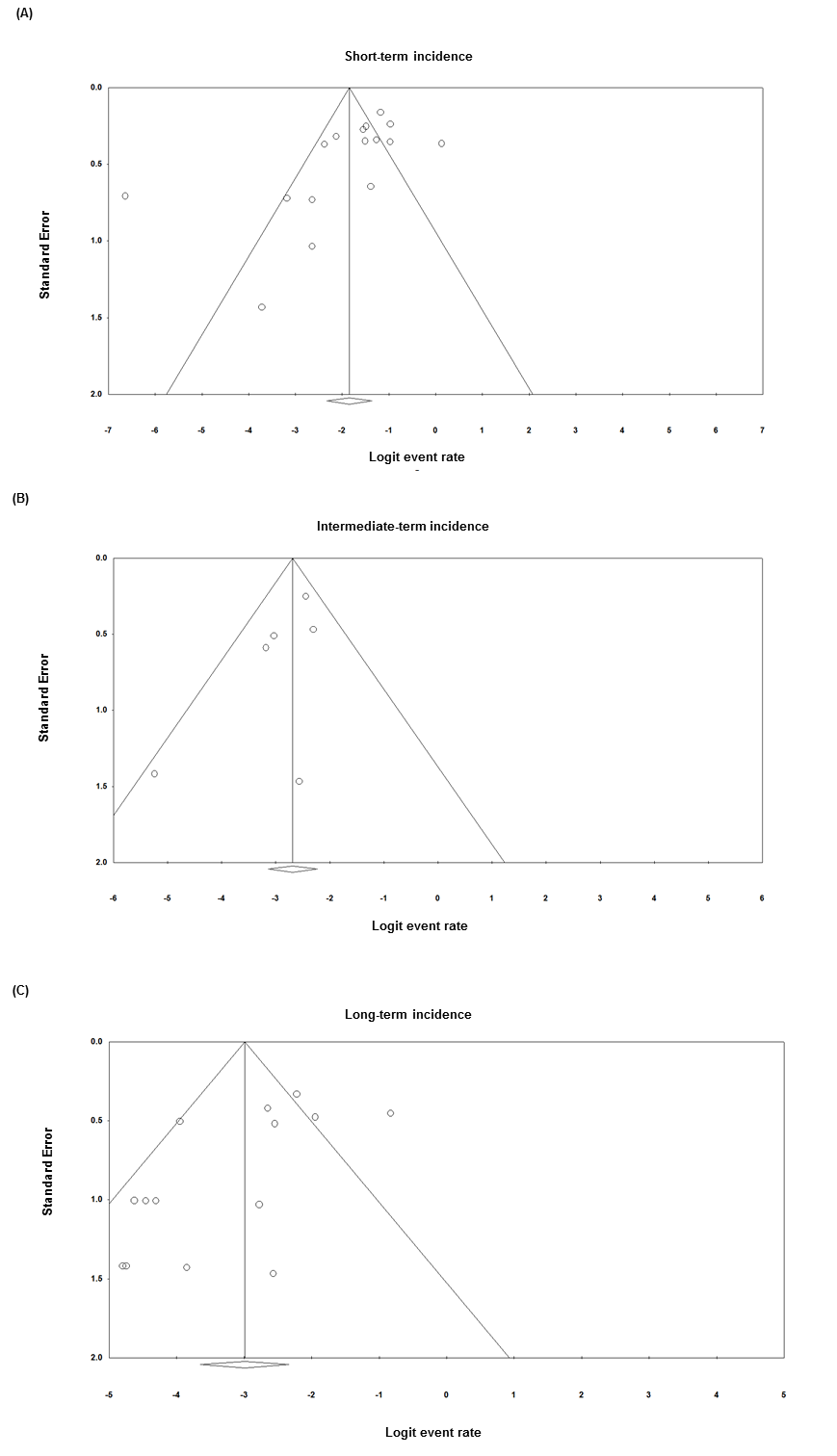

Supplement: S1 Fig — (TIF) [file pone.0154082.s001.tif]
