# Supplementary material for: Systematic Review and Meta-Analysis on Incidence of Altered Sensation of Mandibular Implant Surgery
Source: PLoS One. 2016 Apr 21;11(4):e0154082. doi: 10.1371/journal.pone.0154082 (PMC4839635; doi:10.1371/journal.pone.0154082)
Supplement: S1 Table — (DOCX) [file pone.0154082.s002.docx]

**Appendix Table 1.** PRISMA-P Checklist (Moher et al. *Systematic Reviews* 2015, 4:1)

| **Section/topic**   \|  \| \| --- \| | **Item #** | **Checklist item** | **Remark** |
| --- | --- | --- | --- | --- |
| **ADMINISTRATIVE INFORMATION** | | | |
| Title  Identification | 1a | Identify the report as a protocol of a systematic review | Title |
| Update | 1b | If an update of a previous systematic review, identify as such | Introduction P.3 |
| Registration | 2 | If registered, provide the name of the registry (e.g. PROSPERO) and registration number | Materials and Methods P.5 |
| Authors  Contact | 3a | Provide name, institutional affiliation, e-mail and physical mailing address of all protocol authors | Title page |
| Contributions | 3b | Describe contributions of protocol authors; state guarantor of the protocol. | Please see Statement Authorship Form for author’s contribution.  Chia-shu Lin is the guarantor. |
| Amendments | 4 | If the report represents an amendment of a previously published protocol, identify as such; otherwise state how amendments will be dealt with | The report does not represent an amendment of a previously published protocol. |
| Support  Sources | 5a | Provide sources and types of financial or other support for the review | Acknowledges P.18 |
| Sponsor | 5b | Provide name and contact information for the review sponsor | Acknowledges P.18 |
| Role of  sponsor/funder | 5c | Describe roles of funder(s), sponsor(s) and/or institution(s), if any, in developing the protocol; the decision to submit the protocol for publication and any planned role in the review, including who will have ultimate authority over each of these activities | Acknowledges P.18 |
| **INTRODUCTION** | | | |
| Rationale | 6 | Describe the rationale for the review in the context of what is already known | Introduction P.3-4 |
| Objectives | 7 | Provide an explicit statement of the question(s) the review will address with reference to participants, interventions, comparators and outcomes (PICO) | Introduction P.4-5 |
| Eligibility criteria | 8 | Specify the study characteristics (e.g., PICO, study design, setting, length of follow-up) to be used as criteria for eligibility for the review, giving rationale | Materials and Methods P.5-6 |
| **METHODS** | | | |
| Information sources | 9 | Describe all intended information sources (e.g., electronic databases, contact with study authors, trial registers or other grey literature sources) with dates of coverage | Materials and Methods P.6 |
| Search strategy | 10 | Present draft of search strategy to be used for at least one electronic database, including planned limits, such that it could be repeated | Materials and Methods P.6 |
| Study records  Management | 11a | Describe the mechanism(s) that will be used to manage records and data throughout the review | Materials and Methods P.6-7 |
| Selection process | 11b | State the process that will be used for selecting studies (e.g. two independent reviewers) through each phase of the review (i.e. screening, eligibility and inclusion in meta-analysis) | Materials and Methods P.7 (Study selection, data extraction and assessment of risk of bias were independently performed by the authors CSL and SYW.) |
| Data collection process | 11c | Describe planned method of extracting data from reports (e.g., piloting forms, done independently, in duplicate), any processes for obtaining and confirming data from investigators | Materials and Methods P.7 |
| Data items | 12 | List and define all variables for which data will be sought (e.g., PICO items, funding sources), any pre-planned data assumptions and simplifications | Materials and Methods P.7-8 |
| Outcomes and prioritization | 13 | List and define all outcomes for which data will be sought, including prioritization of main and additional outcomes, with rationale | Materials and Methods P.7 |
| Risk of bias in individual studies | 14 | Describe anticipated methods for assessing risk of bias of individual studies, including whether this will be done at the study or outcome level; state how this information will be used in data synthesis, if planned | Materials and Methods P.8 (Assessment of Study Quality and Risk of Bias) |
| Data Synthesis | 15a | Describe criteria under which study data will be quantitatively synthesised | Materials and Methods P.8-9 (Data Synthesis) |
|  | 15b | If data are appropriate for synthesis, describe planned summary measures, methods of handling data and methods of combining data from studies, including any planned exploration of consistency (e.g. I2, kendall’s tau). | Materials and Methods P.8-10 |
|  | 15c | Describe any proposed additional analyses (e.g., sensitivity or subgroup analyses, meta-regression) | Materials and Methods P.9 (9meta-regression) |
|  | 15d | If quantitative synthesis is not appropriate, describe type of summary planned | N/A |
| Meta-bias(es) | 16 | Specify any planned assessment of meta-bias(es) (e.g., publication bias across studies, selective reporting within studies) | Materials and Methods P.10 (Publication Bias) |
| Confidence in cumulative evidence | 17 | Describe how confidence in cumulative evidence will be assessed (e.g., GRADE), if planned | Materials and Methods P.9 (Assessment of Study Quality and Risk of Bias) |
