# Supplementary material for: Systematic Review and Meta-Analysis on Incidence of Altered Sensation of Mandibular Implant Surgery
Source: PLoS One. 2016 Apr 21;11(4):e0154082. doi: 10.1371/journal.pone.0154082 (PMC4839635; doi:10.1371/journal.pone.0154082)
Supplement: S2 Table — (DOCX) [file pone.0154082.s003.docx]

**Appendix Table 2.** Reasons for Article Exclusion

| Year | First Author | Journal Name | Reason (classification) |
| --- | --- | --- | --- |
| 2015 | Hassani A | J Oral Implantol | Nerve mobilization |
| 2013 | Fernández Díaz JÓ | Int J Oral Maxillofac Surg | Nerve mobilization |
| 2013 | Khajehahmadi S | Int J Oral Maxillofac Surg | Nerve mobilization |
| 2013 | Lorean A | Int J Oral Maxillofac Surg | Nerve mobilization |
| 2010 | Bovi M | Int J Periodontics Restorative Dent | Nerve mobilization |
| 2010 | Hashemi HM | Int J Oral Maxillofac Surg | Nerve mobilization |
| 2005 | Ferrigno N | Int J Oral Maxillofac Implants | Nerve mobilization |
| 2002 | Morrison A | J Can Dent Assoc | Nerve mobilization |
| 2002 | Peleg M | Int J Oral Maxillofac Implants | Nerve mobilization |
| 1999 | Nocini PF | Clin Oral Implants Res | Nerve mobilization |
| 1997 | Kan JY | Int J Oral Maxillofac Implants | Nerve mobilization |
| 1995 | Hirsch JM & Brånemark PI | Br J Oral Maxillofac Surg | Nerve mobilization |
| 1994 | Jensen J | J Oral Maxillofac Surg | Nerve mobilization |
| 1992 | Friberg B | Int J Periodontics Restorative Dent | Nerve mobilization |
| 1992 | Rosenquist B | Int J Oral Maxillofac Implants | Nerve mobilization |
| 2013 | Marianetti TM | J Craniofac Surg | Distraction osteogenesis |
| 2012 | Perdijk FB | Int J Oral Maxillofac Surg | Distraction osteogenesis |
| 2012 | Zwetyenga N | Oral Surg Oral Med Oral Pathol Oral Radio | Distraction osteogenesis |
| 2008 | González-García A | Int J Oral Maxillofac Implants | Distraction osteogenesis |
| 2008 | Günbay T | Oral Surg Oral Med Oral Pathol Oral Radiol Endod | Distraction osteogenesis |
| 2008 | Raghoebar GM | Int J Oral Maxillofac Implants | Distraction osteogenesis |
| 2007 | Perdijk FB | Int J Oral Maxillofac Surg | Distraction osteogenesis |
| 2007 | Saulacić N | J Oral Maxillofac Surg | Distraction osteogenesis |
| 2005 | Enislidis G | Oral Surg Oral Med Oral Pathol Oral Radiol Endod | Distraction osteogenesis |
| 2013 | Juodzbalys G | Clin Oral Implants Res | Injury |
| 2012 | Mundinger GS | Plast Reconstr Surg. | Injury |
| 2011 | Renton T | J Orofac Pain. | Injury |
| 2010 | Froum SJ | Compend Contin Educ Dent. | Injury |
| 2010 | Park JH, | Int J Prosthodont | Injury |
| 2008 | Farole A | J Oral Maxillofac Surg. | Injury |
| 2008 | Kalladka M | Oral Surg Oral Med Oral Pathol Oral Radiol Endod. | Injury |
| 2007 | Tay AB | Int J Oral Maxillofac Surg. | Injury |
| 2014 | Park KR | J Craniomaxillofac Surg. | Osteotomy |
| 2013 | Ueki K | J Craniomaxillofac Surg. | Osteotomy |
| 2012 | Ueki K | J Craniomaxillofac Surg. | Osteotomy |
| 2011 | Bormann KH | J Oral Maxillofac Surg. | Osteotomy |
| 2010 | Pelo S | Int J Oral Maxillofac Surg. | Osteotomy |
| 2008 | Cheung LK | Int J Oral Maxillofac Surg. | Osteotomy |
| 2005 | Kallela I | Oral Surg Oral Med Oral Pathol Oral Radiol Endod | Osteotomy |
| 2015 | Resnik RR & Misch CE | Dent Today. | Review |
| 2014 | Vetromilla BM | Int J Oral Maxillofac Surg | Review |
| 2013 | Barrachina-Díez JM | Int J Oral Maxillofac Implants. | Review |
| 2013 | Chrcanovic BR | Oral Maxillofac Surg. | Review |
| 2013 | Gunarajah DR | J Oral Maxillofac Surg. | Review |
| 2013 | Wanner L | Quintessence Int. | Review |
| 2012 | Kehoe S | Injury. | Review |
| 2012 | Papaspyridakos P | J Dent Res. | Review |
| 2011 | Bagheri SC | Atlas Oral Maxillofac Surg Clin North Am. = | Review |
| 2011 | Palma-Carrió C | Med Oral Patol Oral Cir Bucal. | Review |
| 2010 | Alhassani AA | J Oral Implantol. | Review |
| 2010 | Misch CE | Implant Dent. | Review |
| 2010 | Renton T. | Dent Update. | Review |
| 2009 | Poort LJ | J Oral Maxillofac Surg. | Review |
| 2008 | Greenstein G | J Periodontol. | Review |
| 2006 | Clark GT | J Calif Dent Assoc. | Review |
| 2006 | Greenstein G | J Periodontol. | Review |
| 2006 | Hegedus F | Int J Oral Maxillofac Implants. | Review |
| 2003 | Mraiwa N | Clin Implant Dent Relat Res. 2003;5(4):219-25.. | Review |
| 2002 | Berglundh T | J Clin Periodontol. | Review |
| 2002 | Coulthard P | Cochrane Database Syst Rev. | Review |
| 2002 | Friedlander AH | J Am Dent Assoc. | Review |
| 2002 | Kraut RA | J Am Dent Assoc. | Review |
| 2001 | Ziccardi VB | Atlas Oral Maxillofac Surg Clin North Am. | Review |
| 1998 | Dao TT | Int J Prosthodont. | Review |
| 1998 | Goodacre CJ | J Prosthet Dent. | Review |
| 1995 | Delcanho RE | J Prosthet Dent. | Review |
| 2002 | Marx RE | J Oral Maxillofac Surg. | Transmandibular surgery |
| 1991 | Bosker H | J Oral Maxillofac Surg. | Transmandibular surgery |
| 2011 | Hansen EJ | Eur J Oral Implantol. | Full text not available |
| 2001 | Freeman C | Eur J Prosthodont Restor Dent. | Full text not available |
| 2001 | Louis PJ | Atlas Oral Maxillofac Surg Clin North Am. | Full text not available |
| 2001 | Sullivan DY | Compend Contin Educ Dent. | Full text not available |
| 1998 | Garg AK | Pract Periodontics Aesthet Dent. | Full text not available |
| 1998 | Onstad MS | Dent Implantol Update. | Full text not available |
| 1997 | Heydenrijk K | Eur J Prosthodont Restor Dent. | Full text not available |
| 2015 | Nogami S | Pain Med. | Bone harvesting surgery |
| 2014 | Laino L | BioMed Research International, | Bone harvesting surgery |
| 2013 | Carlsen A | Implant Dent. | Bone harvesting surgery |
| 2013 | Peñarrocha-Diago M | Int J Oral Maxillofac Implants. | Bone harvesting surgery |
| 2012 | Nóia CF | Implant Dent. | Bone harvesting surgery |
| 2008 | Hwang KG | J Periodontol. | Bone harvesting surgery |
| 2008 | Pommer B | Clin Oral Implants Res. | Bone harvesting surgery |
| 2007 | Raghoebar GM | Int J Oral Maxillofac Implants. | Bone harvesting surgery |
| 2006 | Silva FM | Implant Dent. | Bone harvesting surgery |
| 2005 | Schuler R | J Periodontol. | Bone harvesting surgery |
| 2005 | Schwartz-Arad D | Implant Dent. | Bone harvesting surgery |
| 2005 | von Arx T | Clin Oral Implants Res. | Bone harvesting surgery |
| 2001 | Nkenke E | Clin Oral Implants Res. | Bone harvesting surgery |
| 2001 | Raghoebar GM | Clin Oral Implants Res. | Bone harvesting surgery |
| 1991 | el Deeb M | J Oral Maxillofac Surg. | Bone harvesting surgery |
| 2014 | Bertl K | Clin Oral Investig. | Post-mortem |
| 2014 | Esposito M | Eur J Oral Implantol | Same cphort as Felice 2009 |
| 2014 | Felice P | Eur J Oral Implantol | Same cphort as Felice 2009 |
| 2014 | Nicolielo LF | Surg Radiol Anat. | Maxilla only |
| 2014 | Soehardi A | Int J Oral Maxillofac Surg. | Time point was not clearly defined |
| 2014 | Streckbein P | J Craniomaxillofac Surg. | Autologous pressfit bone cylinders and micro-lag-screwfixation |
| 2013 | Anitua E | Int J Oral Maxillofac Implants. 2013 | Drilling tech |
| 2013 | Ebadian AR | J Basic Clin Physiol Pharmacol. | Peri-implantitis |
| 2013 | Givol N | J Periodontol. | Medicolegal cases |
| 2013 | Hawley K | Otolaryngol Head Neck Surg. | Hearing |
| 2013 | Massey ND | J Can Dent Assoc. | Post-mortem |
| 2013 | Ogata Y | Int J Oral Maxillofac Implants. | Flap surgery |
| 2013 | Sammartino G | J Periodontol. | Simulation |
| 2013 | Vázquez-Morales DE | Oral Surg Oral Med Oral Pathol Oral Radiol. | Fracture |
| 2012 | Aynehchi BB | Otolaryngol Head Neck Surg. | Mentoplasty |
| 2012 | Cusack SL | Journal of pain research | Gynecological surgery |
| 2012 | Deppe H | Quintessence Int. | Sinus augmentation |
| 2012 | Tsukiyama Y | J Oral Rehabil. | Occlusal dysaesthesia |
| 2011 | Albu S | American Journal of Rhinology and Allergy | Chronic odontogenic sinusitis |
| 2011 | Al-Nawas B | Int J Oral Maxillofac Implants. | Tube-in-tube implant |
| 2011 | Başa O | Gerodontology. | CT scan |
| 2011 | Froum S | J Periodontol. | CT scan |
| 2011 | Givol N | Oral Surg Oral Med Oral Pathol Oral Radiol Endod. | Medicolegal cases |
| 2011 | Zoghbi SA | J Oral Maxillofac Surg. | Paresthesia as criteria |
| 2010 | Bhatt K | J Oral Maxillofac Surg. | Bioresorbable fixation |
| 2010 | Deer TR | Clin J Pain. | Neurostimulation |
| 2010 | Lethaus B & Verdonck HW | J Oral Rehabil. | Maxillectomy |
| 2010 | Roman JD. | J Minim Invasive Gynecol. | Endometriosis |
| 2010 | Yekta SS | J Oral Maxillofac Surg. | QST |
| 2009 | Triplett RG | J Oral Maxillofac Surg. | Sinus augmentation |
| 2008 | Sammartino G | J Periodontol. | Simulation |
| 2007 | Almas K | Odontostomatol Trop. | TMJ |
| 2007 | Hu KS | J Oral Maxillofac Surg. | Anatomy |
| 2007 | Karabuda ZC | J Periodontol. | Medication |
| 2007 | Libersa P | J Oral Maxillofac Surg. | Sample size unclear |
| 2006 | Abu Hantash RO | Clin Oral Implants Res. | Psychological impact |
| 2005 | Cranin AN | J Oral Implantol. | No abstract |
| 2005 | Goené R | Implant Dent. | Short implant |
| 2005 | Honda MJ | Nagoya J Med Sci. | Facial prothesis |
| 2005 | Iezzi G | J Oral Implantol. | Implant removal |
| 2005 | Khadra M | Swed Dent J Suppl. | Tissue healing |
| 2005 | Schwartz-Arad D | J Periodontol. | Reconstruction |
| 2005 | Yerit KC | Plast Reconstr Surg. | Fracture |
| 2004 | Joshi A | Br Dent J. | Chin graft surgery |
| 2004 | Levin L | Int J Oral Maxillofac Implants. | Sinus lift operations |
| 2004 | Lind G | Eur J Pain. | Neurostimulation |
| 2003 | Chiapasco M | Clin Implant Dent Relat Res. | No report on altered sensation |
| 2003 | Clavero J | Clin Implant Dent Relat Res. | Sinus augmentation |
| 2002 | Balaji SM | Implant Dent. | Maxilla only |
| 2002 | Chaushu G | Int J Oral Maxillofac Implants. | Medicolegal cases |
| 2002 | Fenlon MR | Clin Oral Implants Res. | Psychological impact |
| 2001 | Heller AA | J Oral Implantol. | Injection |
| 2001 | Hori M | J Oral Sci. | Cancer patients |
| 1998 | Piattelli A | Biomaterials. | Implant removal |
| 1997 | Eyrich GK | J Oral Maxillofac Surg. | Fracture |
| 1997 | Flint PW | Otolaryngol Head Neck Surg. | Laryngeal paralysis |
| 1995 | Higuchi KW | J Oral Maxillofac Surg | Derived from the patient cohort of van Steenberg et al. 1990 |
| 1995 | Humphris GM | Int J Oral Maxillofac Implants. | Psychological impact |
| 1992 | Quinn PD | Int J Oral Maxillofac Implants. | Extraoral surgery |
| 1991 | Haers PE | Int J Oral Maxillofac Surg. | Ridge augmentation |
| 2014 | Fernández H | J Oral Maxillofac Surg. | Zygomatic or orbital surgery |
| 2013 | Polligkeit J | J Craniomaxillofac Surg. | Zygomatic or orbital surgery |
| 2012 | Gierloff M | J Craniofac Surg. | Zygomatic or orbital surgery |
| 2012 | Pau M | J Craniofac Surg. | Zygomatic or orbital surgery |
| 2011 | Kruschewsky Lde S | Journal of craniofacial surgery | Zygomatic or orbital surgery |
| 2011 | Singh V | Otolaryngol Head Neck Surg. | Zygomatic or orbital surgery |
| 2010 | Gerbino G | J Oral Maxillofac Surg. | Zygomatic or orbital surgery |
| 2007 | Tay AB | Int J Oral Maxillofac Surg. | Zygomatic or orbital surgery |
| 2006 | Al-Sukhun J | J Oral Maxillofac Surg. | Zygomatic or orbital surgery |
| 2002 | Villarreal PM | Plast Reconstr Surg. | Zygomatic or orbital surgery |
| 2001 | Aitasalo K | J Oral Maxillofac Surg. | Zygomatic or orbital surgery |
| 1997 | Wider TM | Plast Reconstr Surg. | Zygomatic or orbital surgery |
