# Supplementary material for: Systematic Review and Meta-Analysis on Incidence of Altered Sensation of Mandibular Implant Surgery
Source: PLoS One. 2016 Apr 21;11(4):e0154082. doi: 10.1371/journal.pone.0154082 (PMC4839635; doi:10.1371/journal.pone.0154082)
Supplement: S3 Table — (DOCX) [file pone.0154082.s004.docx]

**Appendix Table 3.** Results of Assessment of Study Quality and Risk of Bias

| **Study ID** | **Total score** | **(1)** | **(2)** | **(3)** | **(4)** | **(5)** | **(6)** | **(7)** |
| --- | --- | --- | --- | --- | --- | --- | --- | --- |
| 1 | 6 | 1 | 0 | 1 | 1 | 1 | 1 | 1 |
| 2 | 2 | 0 | 1 | 0 | 1 | 0 | 0 | 0 |
| 3 | 6 | 1 | 1 | 1 | 1 | 0 | 1 | 1 |
| 4 | 4 | 1 | 1 | 1 | 0 | 0 | 1 | 0 |
| 5a | 4 | 1 | 0 | 1 | 0 | 0 | 1 | 1 |
| 5b | 4 | 1 | 0 | 1 | 0 | 0 | 1 | 1 |
| 6a | 4 | 1 | 0 | 1 | 0 | 0 | 1 | 1 |
| 6b | 4 | 1 | 0 | 1 | 0 | 0 | 1 | 1 |
| 7 | 0 | 0 | 0 | 0 | 0 | 0 | 0 | 0 |
| 8 | 4 | 0 | 1 | 1 | 1 | 1 | 0 | 0 |
| 9 | 4 | 1 | 1 | 0 | 1 | 1 | 0 | 0 |
| 10 | 5 | 0 | 1 | 1 | 1 | 0 | 1 | 1 |
| 11 | 5 | 1 | 1 | 1 | 0 | 0 | 1 | 1 |
| 12 | 2 | 0 | 0 | 0 | 1 | 0 | 0 | 1 |
| 13 | 3 | 0 | 0 | 0 | 1 | 0 | 1 | 1 |
| 14 | 5 | 1 | 0 | 1 | 1 | 1 | 1 | 0 |
| 15 | 5 | 0 | 1 | 1 | 1 | 1 | 1 | 0 |
| 16 | 3 | 0 | 1 | 1 | 0 | 0 | 0 | 1 |
| 17 | 4 | 0 | 1 | 1 | 0 | 0 | 1 | 1 |
| 18 | 7 | 1 | 1 | 1 | 1 | 1 | 1 | 1 |
| 19 | 6 | 1 | 0 | 1 | 1 | 1 | 1 | 1 |
| 20 | 3 | 0 | 1 | 0 | 1 | 1 | 0 | 0 |
| 21 | 4 | 0 | 1 | 1 | 1 | 1 | 0 | 0 |
| 22 | 5 | 1 | 1 | 1 | 0 | 0 | 1 | 1 |
| 23 | 5 | 0 | 1 | 1 | 1 | 0 | 1 | 1 |
| 24 | 4 | 0 | 1 | 1 | 0 | 1 | 1 | 0 |
| 25 | 5 | 1 | 1 | 1 | 0 | 0 | 1 | 1 |
| 26 | 3 | 0 | 1 | 1 | 0 | 0 | 0 | 1 |
| Mean | 4.1 | 50% | 64% | 79% | 54% | 36% | 68% | 64% |
